# Novel design and controls for focused DNA microarrays: applications in quality assurance/control and normalization for the Health Canada ToxArray™

**DOI:** 10.1186/1471-2164-7-266

**Published:** 2006-10-19

**Authors:** Carole L Yauk, Andrew Williams, Sherri Boucher, Lynn M Berndt, Gu Zhou, Jenny L Zheng, Andrea Rowan-Carroll, Hongyan Dong, Iain B Lambert, George R Douglas, Craig L Parfett

**Affiliations:** 1 Mutagenesis Section, Environmental and Occupational Toxicology Division, Safe Environments Program, Health Canada, Ottawa, Ontario, K1A 0L2, Canada; 2 Biostatistics and Epidemiology Division, Safe Environments Program, Health Canada, Ottawa, ON, K1A 0K9, Canada; 3 Department of Biology, Carleton University, 1125 Colonel By Drive, Ottawa, ON, Canada, K1S 5B6

## Abstract

**Background:**

Microarray normalizations typically apply methods that assume absence of global transcript shifts, or absence of changes in internal control features such as housekeeping genes. These normalization approaches are not appropriate for focused arrays with small sets of genes where a large portion may be expected to change. Furthermore, many microarrays lack control features that can be used for quality assurance (QA). Here, we describe a novel external control series integrated with a design feature that addresses the above issues.

**Results:**

An EC dilution series that involves spike-in of a single concentration of the *A. thaliana *chlorophyll synthase gene to hybridize against spotted dilutions (0.000015 to 100 μM) of a single complimentary oligonucleotide representing the gene was developed. The EC series is printed in duplicate within each subgrid of the microarray and covers the full range of signal intensities from background to saturation. The design and placement of the series allows for QA examination of frequently encountered problems in hybridization (e.g., uneven hybridizations) and printing (e.g., cross-spot contamination). Additionally, we demonstrate that the series can be integrated with a LOWESS normalization to improve the detection of differential gene expression (improved sensitivity and predictivity) over LOWESS normalization on its own.

**Conclusion:**

The quality of microarray experiments and the normalization methods used affect the ability to measure accurate changes in gene expression. Novel methods are required for normalization of small focused microarrays, and for incorporating measures of performance and quality. We demonstrate that dilution of oligonucleotides on the microarray itself provides an innovative approach allowing the full dynamic range of the scanner to be covered with a single gene spike-in. The dilution series can be used in a composite normalization to improve detection of differential gene expression and to provide quality control measures.

## Background

High-density genomic tools, such as DNA microarrays, provide an important opportunity to study the global response of genomes to particular stressors or conditions. Unfortunately, commercially-available DNA microarrays exhibit several disadvantages when applied to toxicological investigations. For example, in toxicology, the lack of representation of toxicologically-relevant genes on commercial microarrays is an important problem. Statistical issues arising from existing commercial array designs present additional limitations for toxicogenomics experiments. On high-density commercial arrays, the thousands of genes that are not involved in toxic response pathways may remain relatively stable across exposures, thereby weakening the statistical power necessary to identify responsive elements of the genome [1]. Conversely, focused arrays that contain a limited number of features associated with particular biochemical pathways often lack technical and biological control features, such as gene replicates, quality assurance controls and/or normalization features, that are required for experiments in which a large proportion of genes may exhibit differential expression. A final impediment of commercial arrays in applications such as toxicogenomics is their high cost, which hinders the ability to conduct experiments with sufficient numbers of biological samples/replicates. As a result, the use of custom-made focused microarrays has become a popular alternative, allowing the study of genes relevant to a specific hypothesis.

To address the limitations of commercial arrays in toxicogenomics investigations, we have developed a custom mouse oligonucleotide microarray, the HC ToxArray™, which, by virtue of including genes that respond to a variety of chemical and physical stressors, is more relevant for toxicological studies. Furthermore, we have incorporated an extensive series of controls useful for both quality control and normalization of small focused arrays. Typical microarray analyses rely on normalization methods that assume absence of global transcript shifts, or that use internal control features such as housekeeping genes (e.g., [2]). These normalization approaches are not appropriate for focused arrays with small sets of genes [3,4]. Global unbalanced changes are expected due to small array size and gene selection based on pathways predicted to be involved in a particular response. Furthermore, chemical exposures or other parameters (e.g., phase of growth, developmental stage, tissue, disease, etc.) may affect the stability of housekeeping gene expression [5,6]. Therefore, innovative normalization methods are required for these focused arrays (e.g.,)[4,7-9]). The issue of performance measures and quality control standards must be addressed if microarrays are to be applied as tools in regulatory toxicology and risk assessment.

In this study, we describe novel array design features that address the above issues. Specially positioned internal and external control (EC) series on ToxArray™ were developed and characterized in order to eliminate the effects of these biological variables as well as reduce additional technical sources of variability, including uneven hybridization artifacts, minimal spot replication and print tip carryover. Sensitivity of the array spots to low-abundance RNA species was also estimated. Validation of the EC series for normalization and comparison with another common normalization strategy was achieved by determining the gene expression profile in mouse liver samples following treatment with the hepatotoxin phenobarbital.

## Results

### Description and positioning of control spots

**Figure 1:**
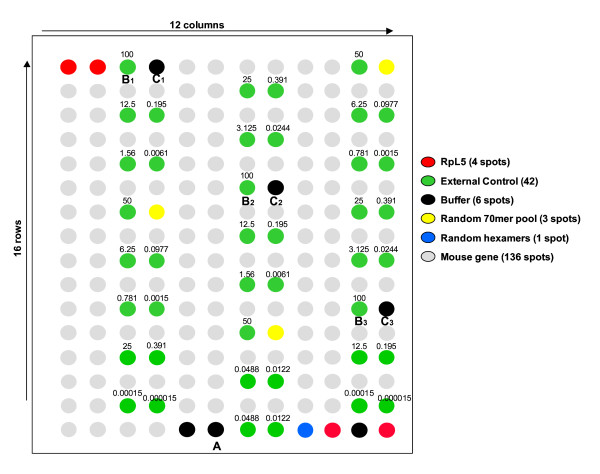
Schematic of the design of one sub-grid of the ToxArray™. Each sub-grid is printed in quadruplicate by one pin. Printing proceeds left-to-right across rows and top row to bottom row. Spot A (bottom row) represents a buffer-only negative control that is printed immediately following another buffer-only spot. B_1 _through B_3 _spots are 100 μM *A. thaliana *spots which reach saturation. Spots C_1 _through C_3 _are buffer-only spots that are printed immediately following B spots. The average signals from these spots were used to calculate the amount of cross-spot contamination (relative to Spot A) using the formula: [(C¯−A)/( B¯−A)]×100
 MathType@MTEF@5@5@+=feaafiart1ev1aaatCvAUfKttLearuWrP9MDH5MBPbIqV92AaeXatLxBI9gBaebbnrfifHhDYfgasaacH8akY=wiFfYdH8Gipec8Eeeu0xXdbba9frFj0=OqFfea0dXdd9vqai=hGuQ8kuc9pgc9s8qqaq=dirpe0xb9q8qiLsFr0=vr0=vr0dc8meaabaqaciaacaGaaeqabaqabeGadaaakeaacqGGBbWwcqGGOaakcuqGdbWqgaqeaiabgkHiTiabbgeabjabcMcaPiabc+caViabcIcaOiqbbkeaczaaraGaeyOeI0IaeeyqaeKaeiykaKIaeiyxa0Laey41aqRaeGymaeJaeGimaaJaeGimaadaaa@3E8D@

**Figure 2:**
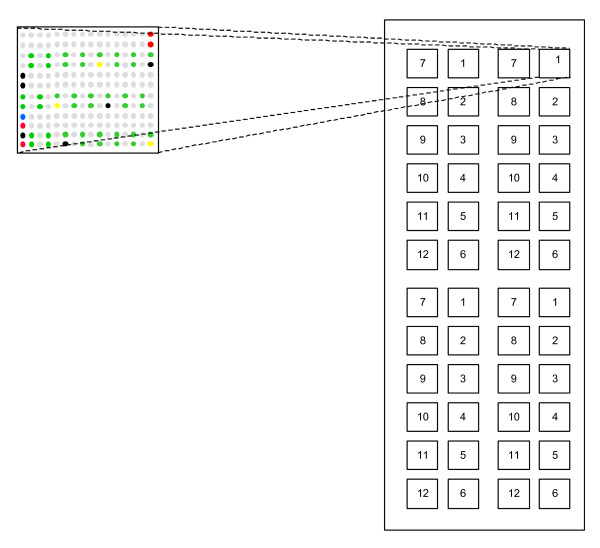
Schematic of the design of one sub-grid and its relative position on a slide of the ToxArray™. Each sub-grid is printed in quadruplicate by one pin. For example, all of the spots in sub grids numbered 1 are printed by the same pin.

We developed an external control (EC) dilution series using the *Arabidopsis thaliana *chlorophyll synthase gene that involves spike-in of a single concentration of the chlorophyll synthase cRNA to hybridize against spotted dilutions (0.000015 to 100 μM) of a single oligonucleotide representing the gene within each sub-grid of the ToxArray™ (Figure 1 and 2; see Methods). Spots of the EC dilutions series were printed in duplicate and in some cases in triplicate. Random dilutions from the series were printed in an evenly spaced pattern within each subgrid, among the oligonucleotide spots representing mouse genes. Several negative control spots, including a random 70 mer pool and randomized hexamers, were included to assess non-specific hybridization. A specific pattern of buffer spots was positioned to allow calculation of cross-spot carryover during printing.

### Evaluation of background, cross-hybridization, signal intensity range and concentration dependence for ToxArray™ external control features

**Figure 3:**
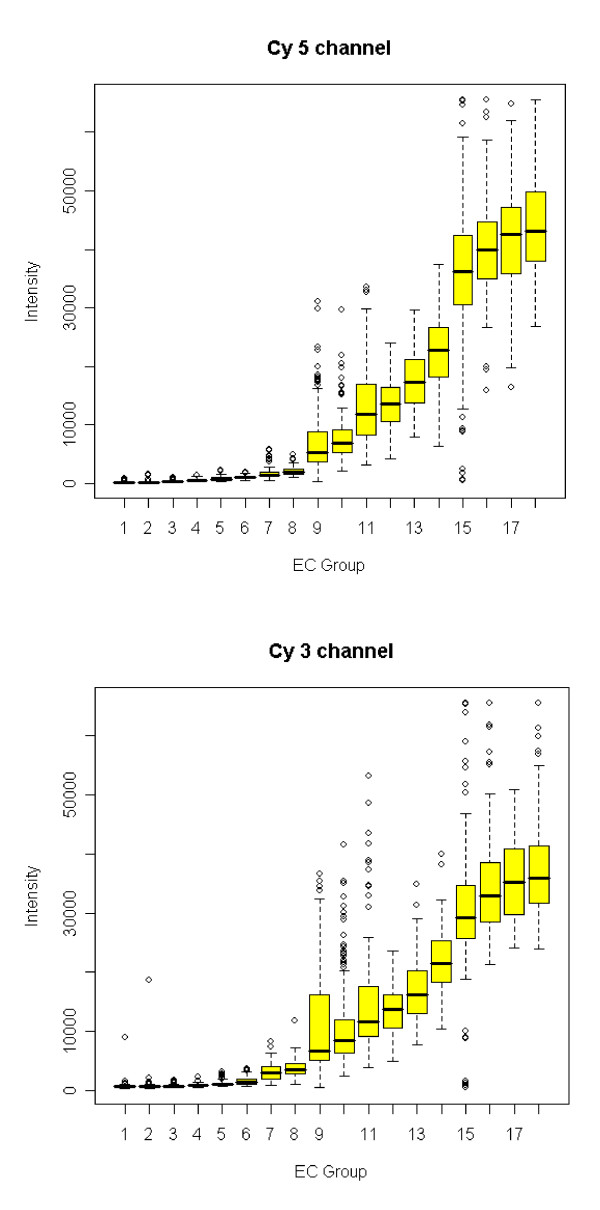
A box plot of signal intensities for each dilution of the *Arabidopsis thaliana *chlorophyll synthase oligonucleotide target on the ToxArray™ is plotted for a typical single hybridization containing experiment and reference samples, each with EC spike-in. Values are raw signal intensities from all spots of each dilution, across all 48 sub-grids. Signal at the greatest dilutions reaches background levels (same intensities as negative controls) and saturation occurs on some spots at the 100 μM dilution.

**Figure 4:**
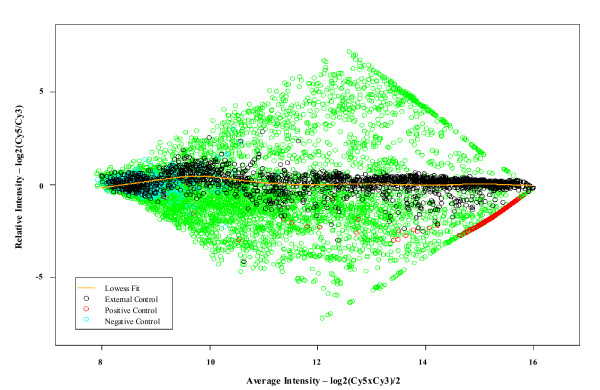
MA plot (log-ratio of the expression intensities versus the mean log-expression of the intensities) for a typical ToxArray™ hybridization containing a mouse liver experimental RNA sample (Cy5-labeled) and Stratagene Universal Reference RNA (Cy3-labeled), each with A. thaliana spike-in control RNA added prior to labelling. The green circles indicate the mouse genes and red circles are the Rpl5 gene which reached scanner saturation in the Cy3 channel at many spots. The EC series covers the full range of signal intensities with no signal gaps. The LOWESS fit shown is through the external controls only, with a span of 0.3.

A BLASTN search of the 70 base oligonucleotide sequence across GENBANK, EMBL, PDB and DDBJ sequence repositories showed significant homologies only with *Arabidopsis thaliana*, *Oryza sativa *and *Zea mays *DNA and RNA sequences. As expected, hybridization of Universal Mouse Reference RNA (Stratagene) or B6C3F1 liver RNA did not result in detectable signal from any of the EC probe dilutions (data not shown). The spotted *A. thaliana *EC dilution series was designed to cover the full range of signal intensities obtained with murine-specific oligonucleotides on the ToxArray™, from background levels to saturation (Figure 3, 4). Standard deviation of the log_2_(Cy5/Cy3) intensity ratios across the range of EC spike-in self vs. self hybridization results obtained in the presence of murine cRNA (Figure 4, black circles) was approximately 0.4 (log_2_), prior to normalization. In comparison to the EC spike-in result, a much larger variance was observed in the log_2_(Cy5/Cy3) intensity ratios typical of a B6C3F1 liver sample vs. Stratagene Universal Reference RNA hybridization result (Figure 4, green circles). The large variation in intensity ratios in the murine cRNA hybridizations were expected to occur due to widely varying abundances of specific mRNAs within these two very different murine RNA sources [10].

**Figure 5:**
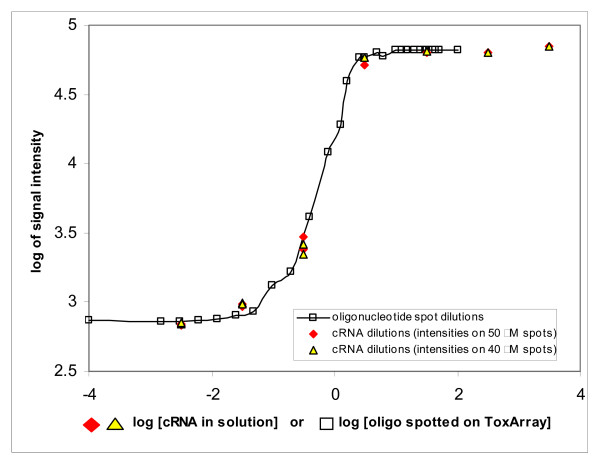
Comparison of effects of dilution of *A. thaliana *spike-in reference RNA in solution (target) and dilution of on-chip, spotted reference oligonucleotide (probe) in mouse cRNA hybridization reactions. Background-subtracted signal intensities are plotted against relative solution cRNA or attached oligonucleotide spot concentrations. Several murine genes with a range of expression levels were employed for slide-to-slide normalization of the spike-in RNA dilution series hybridization results.

A reciprocal dilution experiment was performed to determine the extent to which on-slide concentrations of an oligonucleotide were mimics of the effects of variable concentrations of the solution-phase cRNA partner in bi-molecular hybridization reactions and therefore suitable for normalizing experimental variations in cRNA hybridization. Results from the on-slide dilution series were compared to results from a reciprocal dilution series of the spike-in reference cRNA made in solution. Hybridization of the spotted dilution series with mouse RNA containing a single spike-in concentration of the external reference RNA produced identical profiles of signal intensities compared to the signals obtained from multiple slides hybridized with serial spike-in dilutions of the external reference RNA (Figure 5).

### Estimation of sensitivity of ToxArray™ to low-abundance mRNA species

**Figure 6:**
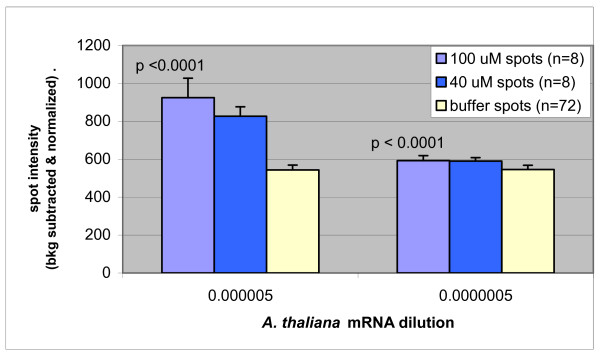
Low abundance sensitivity of the ToxArray™ at the two lowest fractions of *A. thaliana *cRNA in 25 μg of murine cRNA (from dilution data shown in figure 4, above) (p values from t-tests of normalized intensities of *A. thaliana *oligonucleotide and buffer spots). Array-to-array intensities of 40 μM *A. thaliana *spots were normalized to averaged signal from 72 buffer spots, 72 randomized 70 mers, 8 random hexamer spots (40 μM) and eight 40 μM spots of each of three murine genes with raw intensities near that of 40 μM *A. thaliana *spots distributed on each array.

The fractional sensitivity of the ToxArray™ oligonucleotide spots was estimated from hybridizations of the serial dilutions of *A. thaliana *cRNA into a constant amount of cRNA (25 μg) made from the murine polyA+ mRNA, as shown in Figure 5. The smallest fraction tested, 0.0000005 (ng *A. thaliana *cRNA/ng mouse cRNA), gave significantly greater signal intensity than background from buffer-only spots located on the same slide (Figure 6). This level of sensitivity was greater than that necessary to detect one polyA+ mRNA molecule per cell based upon estimates of approximately 300,000 mRNAs per cell [11].

### Detection of positional artifacts using the EC series

**Figure 7:**
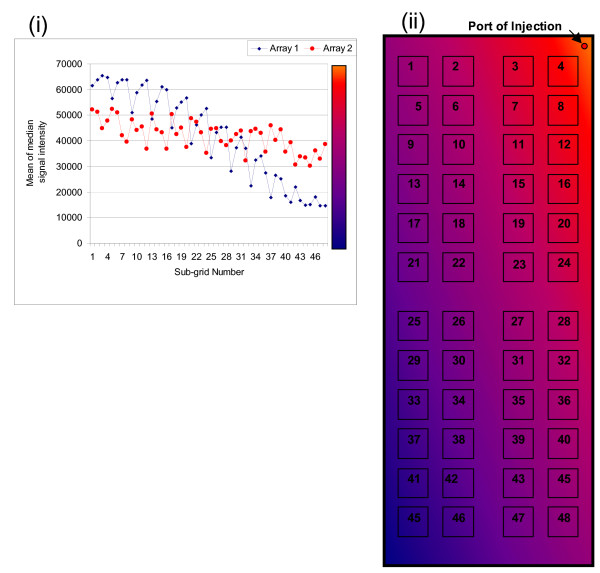
Detection of uneven hybridization using the EC series. An example of the use of external controls for detection of differential hybridization related to the location on the slide relative to the port of injection of hybridization solution on the automated station. (i) The signal intensity of the 100 μM external control is plotted as a function of sub-grid number. Sub-grids are ordered in metacolumns of 4. The higher the sub-grid number, the closer the sub-grid is to the bottom of the slide. Two arrays are plotted. In the automated hybridization station, probe is injected at the top right of each slide (ii). In Array 1, it is noted that the first point dips for every metacolumn, indicating that sub-grids on the left hand side of the slide had lower signal intensities than sub-grids on the right hand side of the slide. Similarly, the sub-grids at the bottom of the slide have lower signal intensity than sub-grids at the top of the slide. In Array 2, the pattern of differential hybridization from the top to the bottom of the slide still exists, but the positional effect is more subtle and therefore would not be detected by visualization of a slide without appropriate controls. These decreasing signal intensities may result from scanner output variables (e.g., photobleaching or optical focus as slide is scanned from top to bottom), in addition to differential hybridization.

**Figure 8:**
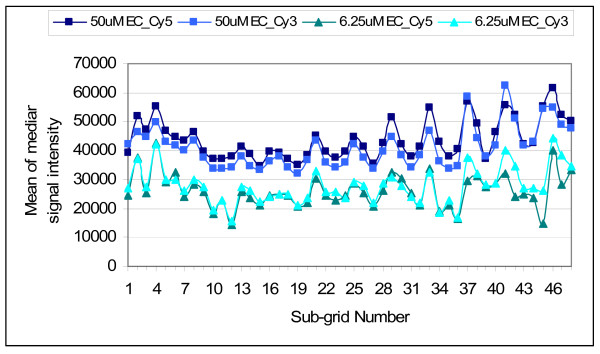
An example of a more typical EC positional hybridization pattern. The array data were derived from a hybridization reaction of one RNA sample included in the EC-normalization validation exercise summarized in Tables 1–3. Cy5 (experiment) and Cy3 (reference) values are plotted for two EC spot concentrations (6.25 and 50 μM) across the 48 subgrids on the array and ordered as shown in Figure 7. A comparison of the average of the Cy5/Cy3 ratios of Grids #10–28 with the combined average of Grids # 1–9 and 11–48 showed no significant difference (t-test, two-tailed, p = 0.390 and p = 0.393 for 50 and 6.25 μM, respectively)

Because each individual on-slide dilution is printed two to three times within each sub-grid, the design allows for a simple evaluation of differential hybridization (grid to grid) on every array. For example, in initial experiments we detected differential hybridization from right to left and top to bottom of our early slides (Figure 7) that was related to the location on the slide relative to the port of injection of hybridization solution on the automated hybridization station. To some extent, the median of the four widely spaced replicate subgrids would normalize less severe systematic end-to-end and side-to-side variations in hybridization intensity that might be produced by automated hybridization station solution injection, since increased hybridization in one area would tend to be compensated by decreased hybridization in another. In the RNA profiling experiment reported in this work, hybridization results with similar defects were excluded, in favor of patterns with less overall difference in intensities across the slide (Figure 8).

### Assessments of negative control spots and print-tip carryover

**Figure 9:**
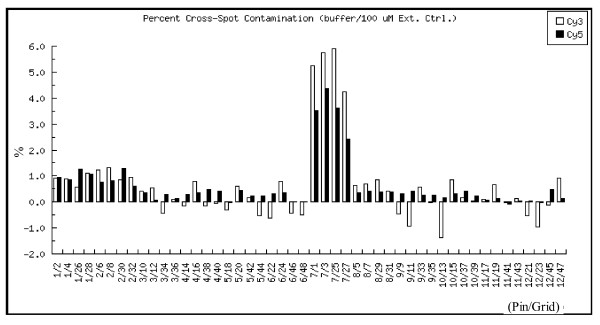
Detection of cross-spot contamination (in %) per pin across a ToxArray™ slide. The slide was hybridized with Cy5-labeled mouse liver RNA and with Cy3-labeled Universal mouse reference RNA. The slide shows a high amount of cross-pin carry-over from the 100 μM EC oligonucleotide to the buffer spot in both the Cy3 and Cy5 channels. Cross-pin carry-over is noted in all sub-grids printed by pin 7.

All three types of negative controls (buffer-only, random 70 mer oligonucleotide pools and random hexamers) showed similar signal intensities within sub-grids indicating: 1) low background signal from the slide substrate and printing process, and 2) that the presence of non-specific oligonucleotides within a spot did not create hybridization artifacts (data not shown). The amount of print-tip carry-over of oligonucleotides from spot-to-spot during the printing-process was determined by considering the signal of buffer spots that were printed prior to (Spot A in Figure 1) and immediately following the saturated signal (Spot C_1 _through C_3 _in Figure 1) associated with the 100 μM *A. thaliana *oligonucleotide (Spot B in Figure 1). Percent cross-spot contamination was calculated by subtracting the signal of the buffer-only spot (Spot A) from the mean of spots printed immediately following a saturated signal (Spots C_1 _though C_3_), divided by the mean signal intensity of the saturated spot (Spots B_1 _though B_3_) minus the buffer-only background (Spot A) (i.e., [C-A]/[B-A] × 100, from Figure 1; grids are printed left to right). A plot showing a chip with a high proportion of cross-spot contamination for several print groups (groups 1, 2, 7 and 8) is shown in Figure 9. Using this measure, the average cross-spot contamination in the separately printed arrays of the PB RNA profiling experiment was 0.60% (± 0.02 standard error) and 0.67% (± 0.02 standard error) for Cy5 and Cy3 respectively.

Two additional estimations of carryover artifact amount and frequency could be made from other spot juxtapositions existing within each subgrid. In the first case, three 50 μM spots were printed prior to three random-70 mer oligonucleotide pool spots. This situation was directly comparable to most of the oligonucleotides representing murine genes, in that carryover from the first spot would be deposited into the following 40 μM spot, rather than buffer. Among arrays employed in the PB experiment described below, we found that the frequency of pins displaying carryover from 50 μM EC spots into the 70 mer pool spots was less (by 2 to 3-fold on a per-pin basis) than the estimate from the 100 μM spot analysis. By design, both 100 μM spots and the 50 μM EC spots gave hybridization signals that were near to, or fully saturated, thus providing optimal levels of signal to determine carryover. There was also a single RpL5 spot printed ahead of a buffer spot in each grid. RpL 5 usually also had very high signal levels that approached or reached signal saturation in most hybridizations, allowing another independent estimation of carryover. Only a single pin displayed carryover in the PB experiment. When measured with the 100 and 50 μM EC spots and RpL5 spot described above, that pin produced 2/3, 2/3, 0/1 individual spots with significant carryover (greater than 0.5% of signal), respectively.

### Comparison of Two Normalization Procedures for Detection of Differential Gene Expression using ToxArray™ and RT-PCR Validation of Presumptive Differential Gene Expression

Male B6C3F1 mice were given oral doses of 100, 10, 1 and 0.1 mg/kg PB or solvent control. Cy-labeled liver cRNA was hybridized against mouse reference cRNA (Stratagene), and MAANOVA was used to examine whether the ToxArray™ could detect both expected and novel differential expression resulting from PB exposure. The first analysis employed a composite LOWESS normalization of microarray intensity values. This approach calculated a LOWESS normalization of the median values of all data in each print-tip group in addition to a LOWESS normalization of the chip EC titration series. A normalization constant that combined these two into an intensity-dependent, weighted average was used [8]. We found a total of 35 genes that were differentially expressed (p < 0.05 using the James-Stein Shrinkage F-Test [12] adjusted for the False Discovery Rate (FDR); Table 1) with respect to the overall treatment effect. These p-values obtained from the Fs-Statistic were estimated using 1000 permutations with residual shuffling. Pairwise comparisons showed that 16 of these genes expressed differential abundances of RNA at the highest dose of PB (100 mg/kg), while 4 were found to be differential at the 10 mg/ml dose. No genes were identified as differentially expressed in pairwise comparisons at the 1 mg/kg dose, nor at the lowest dose (0.1 mg/kg, not shown).

The 16 genes with significant pairwise comparisons after EC normalization were analysed for possible print-tip carryover artifacts, since in the array batch used in the experiment, one of the twelve printing pins had produced carryover effects (see previous section). Four of the 16 genes were located within the suspect print group; however, none of the four oligonucleotides were printed in positions likely to have been affected by carryover effects, such as locations following a differential gene or a gene with very high relative expression.

In a second analysis of the same data, using a standard LOWESS without incorporating the ECs, 39 genes were statistically significant (p < 0.05) after a FDR adjustment based on the James-Stein estimator (Table 2). Between the two analyses, there were 24 genes that were in common (rows with bold text in Table 2), 11 genes specific to the EC-normalized analysis and 15 specific to the LOWESS normalized analysis (none were affected by carryover artifacts).

To select a sub-set of genes for RT-PCR analysis, microarray gene expression data normalized with EC and without EC (Tables 1, 2) were sorted according to the FDR adjusted P-values. A list of genes for each type of normalization was derived, in which p-values less than 0.05 were recorded for the FDR adjusted overall treatment effect **AND **for one of the pairwise comparisons, comparing the treated samples to the control. The union of the two lists consisted of 27 probes, with a total of 31 pairwise comparisons. Two additional probes (Anpep, Cyp2a4; giving four additional pairwise comparisons) were also included, since earlier statistical calculations (1000 permutations with residual shuffling) had indicated both significant pairwise comparisons and overall effects, but these values were not confirmed in the analyses presented in Tables 1 and 2. Results from the microarrays for these 35 overall and pairwise comparisons plus the results from RT-PCR analyses for 27 of these comparisons are presented in Table 3.

The RT-PCR analyses included all 19 pairwise comparisons from the two microarray normalizations that gave conflicting conclusions on whether the respective 17 probes detected differentially expressed mRNAs (Table 3). Based on these 19 comparisons, the results from the normalization using the composite LOWESS (with EC) revealed 15 comparisons that were in agreement with the RT-PCR results, compared to 6 out of 19 based on the results from LOWESS normalized data. Additional RT-PCR analyses were done using RNA samples which gave microarray results (6 probes with 8 pairwise comparisons) that were in agreement between the EC and without EC analyses (Table 3). All six microarray probe results were confirmed to be correct, although two pairwise comparisons for two of the probes conflicted with the RT-PCR results. Results from the RT-PCR were used to determine the true positives, false negatives, false positives and true negatives, and to calculate the sensitivity, specificity and the efficiency of the normalization methods (Tables 4 and 5). Using the EC series (composite normalization) yielded higher sensitivity, specificity and efficiency compared to not using the EC series for normalization, considering either all pairwise comparisons or just those in disagreement.

Two Chi-square contingency table analyses comparing the results from the RT-PCR and the results from both microarray analyses were conducted. Only the 19 probes that were in disagreement between the two microarray results were included and a Yates correction (a continuity correction) was applied. The RT-PCR and the results from the composite LOWESS indicated an association with a p-value of 0.0485, whereas RT-PCR and the results from the LOWESS normalized data without the external controls showed no association (p-value = 0.2301).

### Effect of Spot Replication on Detection of Differential Gene Expression using ToxArray™

The effect of spot replication (spots arranged in 4 replicate supergrids, per array) on the power of ToxArray™ to detect differences in expression was estimated by conducting the EC-based and LOWESS-based normalizations, using one randomly chosen spot per array. The number of genes identified as significantly differentially expressed was reduced in the single-spot analysis to 13 and 16 genes revealed by EC and LOWESS normalization, respectively, compared to 35 and 39, respectively, by the 4-spot analysis. Only eight of the 24 genes identified in common in the 4-spot analysis were also identified in common in the single spot analyses (Cyp2b10, Cyp2b9, Gstm3, Gdf15, Gadd45b, Gadd45a, Lpin1, Por). However, several other genes identified in common by the 4-spot analysis were partitioned into one of the two single-spot analyses: four of eight genes specific to the non-EC single-spot normalization (Gsta4, Rbp1, gstm2, Ephx1) and two of five genes specific to the single-spot EC normalization (Rbp1, AhR). Thus, approximately half of the genes found in common from the 4-spot analyses were found by either single spot analysis. Smaller proportions of genes specific to either normalization in the 4-spot analysis were found to be differential by the single spot analyses: 2/15 for non-EC (Gsta2 Ugt1a6) and 2/11 for EC (Keap1, Bhmt). One gene specific to the single-spot EC normalization and two genes specific to the single-spot non-EC normalization were not identified by the respective 4-spot analyses, reducing the proportion of genes found by the single spot analyses to 0.34 and 0.36 for EC and non-EC, respectively, in this comparison of spot replication effect.

## Discussion

Appropriate quality controls and protocols for assessment of printing and hybridization in microarray experiments are crucial for ensuring high-quality data and establishing performance standards. Despite this, controls used for specifically addressing these issues are rarely reported in microarray publications. In addition, the development of features for normalization within, and between arrays is critical for all microarray applications, and is particularly relevant to the application of small focused arrays where a relatively large portion of the pathways may show changes in gene expression.

We developed an EC dilution series using the spike-in of a single *Arabidopsis *chlorophyll synthase gene hybridized to a series of dilutions of the complementary oligonucleotide printed on the microarray. In contrast with other external spike-in procedures that use a mixture of varying amounts of different control RNAs spiked into total RNA to derive signals across a range of intensities (e.g., [4]), our approach maintains a constant single input of external RNA, while varying the dilution of the EC oligonucleotide on the slide itself. This simple and flexible method allows a more robust quantitation of the spike-in compared to other methods. In addition, the EC series was designed to cover the full range of signal intensities (with no gaps) over the ToxArray™, reaching both background and saturated intensities (Figures 3, 4 and 5). The array incorporates a design that allows the use of the ECs, in combination with negative control spots, for detection of several technical problems that may arise in the printing, hybridization or scanning of a microarray, permitting correction or exclusion during subsequent analyses. Differential intensities of dye fluorescence on microarrays may arise from factors related to physical or chemical properties, differences in the efficiency of incorporation during probe synthesis and as a result of variations in scanner operation. These EC features can be applied in combination with LOWESS to normalize such systematic effects. Dye bias due to differential hybridization that, in part, may result from concentration-dependent effects within or between target cRNA populations and array spots that might arise during hybridization and washing procedures, may also be subject to this type of normalization. In this regard, it has been shown that tethered nucleic acid probes can behave differently depending on the nature of their supports [13], but here it was shown that the hybridization pattern derived from dilution of the EC oligonucleotide on the ToxArray™ slide surface was a close mimic of the pattern of hybridization resulting from similar dilutions of labeled cRNA in solution. A close relationship between these two concentration vs. intensity curves permits subsequent normalization adjustments of variations in murine cRNA hybridization intensity ratios, without introducing large errors. Composite LOWESS adjustments across the range of EC intensities on each array would be likely to represent similar differences in concentration-dependent hybridization amounts of murine genes, across samples.

Subtle unequal hybridization resulting in spatial patterning in dye fluorescence across an array might be undetected by visually examining the arrays, but may be revealed by the presence of increased variances of Cy3/Cy5 ratios among the 48 EC replicates and four gene replicates of each grid. EC features might also be used to control positional and slide-to-slide hybridization effects that are more erratic, such as those resulting from exclusion by bubbles, by taking an average of the ECs within a subgrid, dividing each spot within the subgrid by the EC average, followed by multiplication of each spot by an overall slide average using the external controls. This would be done for each channel separately. Further refinements in normalization can be envisioned that would employ attributes of the EC hybridization curve, other than its average value. A systematic examination of these alternate approaches has not yet been made.

The limited sequence homology of the *A. thaliana *oligonucleotide among the genomes of organisms with published sequence information, suggests possible applications to gene expression studies in a wide variety of organisms. Since oligonucleotide probes designed with constraints on length, G+C% and secondary structure produce similar, parallel hybridization values over large cRNA concentration ranges [14], the choice of oligonucleotide used to produce an EC series in the manner we have described is not necessarily restricted to the sequence employed in the present experiments.

The placement of negative control features within the ToxArray™ design is an innovative approach to allow for the evaluation of cross-spot contamination during the printing process. Negative controls following either a saturated signal or a buffer spot were used to demonstrate the carry-over of oligonucleotides within print-tip groups in a print-run (Figure 9) (this is a frequent problem in microarray printing that is rarely mentioned or quantified) [15]. Carry-over can significantly impact analysis of differential expression if low intensity spots are printed following spots that vary in intensity and occasionally show high signals. Similarly, carryover effects would degrade the usefulness of the EC series. Each print-run should be evaluated to ensure that this effect is minimized, especially with the (demonstrated) high fractional sensitivity of the Toxarray™ (1/2,000,000 transcripts). The knowledge of cross-spot contamination could be incorporated into the selection of genes for RT-PCR confirmation and final analysis of differential gene expression, or print tip groups with unacceptable levels could be removed from the analysis.

Lastly, the ToxArray™ incorporates 4 replicates of each oligonucleotide spot distributed into separate subgrids on each slide. This degree and placement of spot replication can significantly increase the reliability of fluorescence measurements of gene expression levels [11,16] and would thereby decrease variability of two-color ratios. Indeed, fewer genes were identified as differentially expressed based upon single oligonucleotide spots as compared to the four replicates in this study.

We exposed mice to a known hepatotoxin to test the hypothesis that global normalization approaches for focused microarrays will generate a higher proportion of false findings, relative to an approach using external features. Composite normalization incorporating the EC features (described in methods) resulted in a list of 36 genes with adjusted p-values less than 0.05. The predominant pathways represented among the genes that were differentially expressed included stress response, growth differentiation, apoptosis and xenobiotic metabolism. Among the latter, cyp2b10, cyp2b9, Gsta2 and Ephx1 were validated by RT-PCR. The induction of these pathways is consistent with the known pathologic effects of PB. This response was employed to provide confirmation, when verified by RT-PCR, that the composite normalization would score higher, as expected, on all measures of effectiveness, compared to the global normalization approach.

We expect the composite normalization to be even more superior in experiments where larger portions of genes are responsive. Although PB was an effective modifier of gene expression in this study of murine liver, only about 40 genes of the total 1600 on ToxArray™ might have been categorized by RT-PCR as significant responders, predicting from the existing microarray data and the observed frequencies of RT-PCR confirmation. This was a small proportion (2.5%) of genes represented on the array. Increased numbers of responding genes might have been expected, given higher PB doses or a longer treatment period.

The relatively greater effectiveness of the EC composite normalization approach has also been found in a complementary study examining murine liver response to polychlorinated biphenyl exposure, in which 22 genes were validated using RT-PCR (data not shown – to be published elsewhere). The summary statistics presented in this study should be interpreted with care as there was some potential for selection bias in the choices made for RT-PCR validation among the genes identified as differential by both normalization procedures. However, we are currently carrying out additional RT-PCR experiments with other microarray datasets to achieve more representative estimates.

## Conclusion

We demonstrate the application of a simple external control dilution series, in combination with a novel DNA array design, to yield improved detection of differential gene expression and provide quality control measures. The appropriate placement of controls and replication of probe spots allows for quantification of positional hybridization effects and cross-spot contamination. The application of a composite normalization, which incorporates an external control dilution series, results in an improvement in the sensitivity and predictivity of the test, as well as a minimization in false positive detection. Dilution of oligonucleotides on the microarray itself provides an innovative approach allowing the full dynamic range of the scanner to be covered with a single gene spike-in.

## Methods

### ToxArray™ gene selection

The list of ToxArray™ genes was compiled from our own data, as well as extensive information from published studies investigating gene expression changes, assessed using microarrays or other technologies, following toxicant exposures. Mouse genes (and homologues of rat and human genes) shown to be useful in toxicant-specific profiling were included [4,17-19]. Additionally, a large fraction of murine genes known to be involved in DNA repair, as well as the mouse cytochrome p450 family, were included. Our current list includes approximately 1600 genes known to respond to a wide range of toxic stressors. Approximately 50 housekeeping genes have been included on the array. Single-stranded 5'-C6 amino modified 70 mer oligonucleotide probes were designed and synthesized by Qiagen Inc (Alameda, CA, USA).

### ToxArray™ Design

The ToxArray™ consists of 12 sub-grids (16 rows × 12 columns) printed in quadruplicate (for a total of 48 sub-grids). External control (EC) normalization features are present in every sub-grid and consist of a series of 18 dilutions of a single probe corresponding to the *Arabidopsis thaliana *chlorophyll synthase gene (RefSeq NM_115041). The dilution series of the probe is prepared in ArrayIT spotting solution (Telechem International, Sunnyvale, CA, USA) (concentrations range from 0.000015 μM to 100 μM). Each EC probe dilution is printed at least twice per sub-grid (42 EC features per sub-grid) for a total of more than 2000 EC features across the space of the entire array. Each sub-grid also contains three types of negative controls including: buffer-only spots, randomized pools of non-specific 70 mer oligonucleotides (Qiagen Inc.; validated to ensure that they do not match any known mouse genes), and random hexamers (Qiagen, Inc.) in ArrayIT buffer (Figure 1). The latter two negative controls are useful to detect background resulting from fluorescence signal from oligonucleotides or random non-specific binding. Within each sub-grid, placement of one buffer control (Spots C; Figure 1) immediately follows the printing of a 100 μM EC oligonucleotide (Spots B; Figure 1). The 100 μM signal reaches saturation; juxtaposition of the buffer control spot immediately following the saturated control allows the calculation of cross-spot contamination, or carry-over of oligonucleotides across individual spots by the printing pin. Cross-spot contamination was evaluated by comparing the signal from spots C, to a buffer-only spot within the same sub-grid that is printed immediately following another buffer-only spot (Spot A; Figure 1).

A 70 mer oligonucleotide probe was designed and synthesized for the *Arabidopsis thaliana *chlorophyll synthase gene (At3g51820; RefSeq NM_115041) (Qiagen Inc., Alameda, CA, USA). *A. thaliana *chlorophyll synthase RNA was produced through *in vitro *transcription of a plasmid provided by the University Health Network Microarray Centre (Toronto, ON, CA) (the plasmid contains a fragment of the *A. thaliana *chlorophyll synthase gene) using the T7 RiboMAX™ Express Large Scale RNA Production System according to manufacturers protocol (Promega Corporation, Madison, WI, USA). *In vitro *transcribed RNA was quantified by UV light absorbance and visualized for transcript integrity by gel electrophoresis.

### ToxArray™ Production

Oligonucleotides were diluted in ArrayIT Spotting Solution (Telechem International) to 40 μM and printed onto PowerMatrix slides (Full Moon Biosystems, Sunnyvale, CA, USA) with the ChipWriter Pro robotic arrayer (BioRad Laboratories, Mississauga, ON, CA) at 65% relative humidity. Printing pins were washed in water, dried, and sonicated in water three times between each pin loading. Following printing, slides were placed in a humid chamber, containing saturated sodium chloride solution, with a relative humidity of 65–75% for 10–14 hours. Slides were air dried at room temperature for 30 minutes. Prior to hybridization slides were pretreated according to manufacturer's protocol (Full Moon Biosystems). Briefly, slides were incubated at room temperature in 0.2X SSC solution containing 0.2% SDS and 0.1% BSA, preheated to 55°C for 15–30 minutes on an orbital shaker, rinsed thoroughly in Milli-Q water, and dried by centrifugation at 500 rpm for 5 minutes.

### Animal Treatment

Male B6C3F1 mice (age 27–35 days) obtained from Charles River were allowed food and water *ad libitum*. The animals were housed in individual cages under a 12 hr light/12 hr dark lighting schedule, constant temperature and humidity, and provided with an enriched environment. Animals were acclimatized for at least 2 weeks. Treatment groups consisted of 5 animals dosed by oral gavage with 100, 10, 1 or 0.1 mg/kg Phenobarbital (PB) (CAS: 57-30-7) or 0.9% saline (vehicle control group) for 3 consecutive days. Animals were sacrificed four hours after the last PB exposure. Animals were anesthetized with CO_2_, 0.5–0.7 mL of blood was collected by cardiac puncture, and animals were sacrificed by cervical dislocation. Liver was removed, flash-frozen in liquid nitrogen, and stored at -80°C for further analyses. All animal care and handling were in accordance with Canadian Council for Animal Care Guidelines and were reviewed by the Health Canada Animal Care Committee prior to the start of the study.

### RNA isolation

Total RNA from mouse liver was isolated using Trizol Reagent (Gibco-BRL, Life Technologies, Gaithersburg, Maryland, USA) according to the manufacturer's protocol. Briefly, 1 mL of Trizol reagent was added per 50–100 mg tissue and homogenized. Homogenized samples were incubated for 5 minutes at room temperature; chloroform was added at 0.2 mL per 1 mL Trizol reagent, mixed by inversion and incubated for 3 minutes. The aqueous phase was separated by centrifugation, and RNA was precipitated by the addition of isopropanol at 0.5 mL per 1 mL Trizol reagent and incubation for 10 minutes at room temperature. RNA was washed in 75% ethanol and re-dissolved in 1 μL nuclease-free water per 1 mg of tissue. Total RNA was further purified using the RNeasy Mini Kit (Qiagen Inc.) according to the manufacturer's protocol. RNA quality and quantity was assessed by UV spectrophotometry and 28S:18S ratios obtained by Agilent 2100 bioanalyzer (Agilent Technologies, Palo Alto, CA, USA).

### Preparation of labeled cRNA

Labeled cRNA was prepared using the Low Input Fluorescent Linear Amplification Kit from Agilent Technologies according to manufacturer's protocol. For analysis of differential expression following PB exposures, 5 ng of EC RNA was spiked into 5 μg of liver RNA sample and mouse reference RNA (Stratagene, La Jolla, CA, USA). RNA was converted to cDNA using oligo dT-T7 promoter primer and random hexamers. Following second strand cDNA synthesis, cDNA was converted to cRNA and amplified by T7 RNA polymerase in the presence of Cy5- (liver cDNA) or Cy3-CTP (reference cDNA). The amplified cRNA product was purified using the RNeasy Mini Kit (Qiagen, Inc.), re-suspended in nuclease-free water, and quantified by UV spectrophotometry.

### Automated Hybridization Protocol

Automated microarray hybridizations were carried out in an HS 4800 hybridization station (TECAN, Research Triangle Park, NC, USA). Microarrays were pre-washed two times (50°C, 30 minutes; 0.2× SSC, 0.2% SDS, 0.1% BSA). Fifteen μg of labeled Cy5 and Cy3 cRNA probe was fragmented in 25× fragmentation buffer (Agilent *In situ *hybridization kit-plus, Agilent Technologies) at 60°C for 30 minutes. Fragmented cRNA was mixed with 2× hybridization buffer (Agilent *In situ *hybridization kit-plus) and hybridized on the ToxArray™ chips at 60°C for 17 h. Chips were washed with 6 × SSC, 0.005% Triton X-102 at 50°C for 10 min, with 0.1× SSC, 0.005% Triton X-102 at room temperature for 15 min, and rinsed with 0.06× SSC. Microarrays were dried by centrifugation at 500 rpm for 5 minutes. For PB experiments, sample RNA was labeled with Cy5 and Universal mouse RNA was labeled with Cy3 and the automated procedure was used for hybridization and washing of all slides.

### Real time PCR

RT-PCR was used to confirm differential gene expression of transcripts from microarray analyses. Reverse transcription was carried out in a 100 μl reaction mix with SuperScript II (Invitrogen, Burlington, ON, CA) using 1 μg total RNA per animal. Quantitative PCR was performed with an iCycler IQ real-time detection system (BioRad Laboratories) using SYBR-Green. Using 1 μl reverse transcription solution and gene-specific primers (Qiagen, Mississauga, ON, Canada), PCR was performed in a 25 μl reaction Supermix (BioRad Laboratories). Primers were designed using Beacon design 2.0 (Premier BioSoft International, CA, USA) and sequences are available upon request. PCR reactions for each RNA species were performed in duplicate within a single plate, and the values of threshold cycle were averaged. Gene expression levels were normalized to β2-microglobulin, which was found to be stable on the DNA microarrays. PCR efficiency was examined using the standard curve for each gene, determined from reactions that were included in wells of the respective plate. Primer specificity was assured by the melting curve for each gene. A *t*-test was used for statistical evaluation.

### Image analysis and statistics

Arrays were scanned on a ScanArray Express (Perkin Elmer Life Sciences, Woodbridge, ON, Canada). To optimize the dynamic range of microarrays and allow the quantification of both high and low copy expressed genes, the same arrays were scanned using different photomultiplier settings allowing selection of images with saturated image intensities on the 50 and 100 μM EC spots and on oligonucleotide spots for high-abundance genes such as Rpl5. Raw pixel intensities were derived in ImaGene 5.6 (BioDiscovery, Los Angeles, CA) and median signal intensities (not background subtracted) were used for subsequent analyses. Obtained data conformed to the suggested average [log2(Cy3*Cy5)/2 of 10 to 12 (1024 to 4096 RFUs) [20]. Data is available through ArrayExpress [21]. Present calls were determined as signals that were greater than the mean of the random 70 mer oligonucleotide negative controls plus three times the standard deviation (signals within the range of three standard deviations were flagged). Data from all samples were read into SAS 8.2 (SAS Institute Inc, Cary, NC, USA). The data were normalized using the composite LOWESS method [8] which takes a weighted average from a LOWESS fit using only the ECs with a span of 0.3, and a second LOWESS fit for each of the 12 print-tip groups with a span of 0.5. Technical replicates on arrays were collapsed by use of the median normalized relative intensity. For comparison, a second normalization was carried out on PB samples using a LOWESS with a smooth span of 0.4 without incorporation of ECs. The MAANOVA 2.0 [22] library in R was used for graphical displays and analysis. Ratio-intensity plots for each array were obtained. Higher-level statistical analysis, including detection of differential expression was also conducted using MAANOVA methodology [1,23].

## List of Abbreviations

EC: External Control

PB: Phenobarbital

LOWESS: Locally Weighted Scatterplot Smoother

RT-PCR: reverse transcription – polymerase chain reaction

## Authors' contributions

The concept of the ToxArray™ was CLY's. CLY, SB and CLP developed the external control series and the printing design. CLY designed and co-ordinated the PB experiment and drafted the manuscript. SB and CLY curated the original ToxArray™ gene list. SB managed the oligonucleotide library, developed QC/QA approaches, worked on the first draft of the manuscript and participated in the technical variability experiment. GZ & CLP optimized the printing and hybridization of the ToxArray™, printed all arrays used, designed and conducted validation experiments for the on-slide dilution series, determined the fractional sensitivity of the array and modified the draft manuscript. MLB performed the technical variability experiment and image analysis. AW carried out all statistical analysis and experimental design for microarray experiments. JLZ carried out the PB animal experiment and the microarray analysis of the PB experiment as well as the RT-PCR. AR managed and designed oligonucleotides for the ToxArray™ and participated in the design of the ToxArray™. HD conducted experiments to optimize the ToxArray™ printing and hybridization. IBL contributed to the gene list and manuscript writing, supervised and advised SB. GRD participated in the design and coordination of the work, made contributions to the manuscripts and was the overall team leader. CLP and CLY were the project leaders. All authors have read and approved the final manuscript.

## Figures and Tables

**Table 1:** ToxArray™ data normalized using EC: Overall adjusted p after exposure to PB and fold changes (relative to Control) and pairwise p-values at each dose for each gene (probe) that showed differential expression.

			Control versus 100 mg/kg		Control versus 10 mg/kg		Control versus 1 mg/kg		James-Stein F-Test ^†^
			
Gene(probe) Name>	Full name	GenBank	Fold change	Pairwise P-value	Fold change	Pairwise P-value	Fold change	Pairwise P-value	Overall P-value
Cyp2b9	cytochrome P450, 2b9, phenobarbitol inducible, type a,	NM_010000	8.29	0.0000	3.22	0.0000	1.16	0.8087	0.0000
Cyp2b10	Cytochrome P450, 2b10, phenobarbitol inducible, type b	NM_009998	5.10	0.0000	2.95	0.0000	-1.08	0.8437	0.0000
Gdf15	growth differentiation factor 15	NM_011819	2.83	0.0000	1.25	0.3499	1.07	0.8667	0.0000
Gstm3	glutathione S- transferase mu 3 (EC 2.5.1.18)	J03953	2.16	0.0000	1.04	0.8458	1.30	0.4200	0.0000
Lpin1	lipin 1, alias fatty liver dystrophy	NM_015763	-1.94	0.0009	-1.18	0.3932	-1.08	0.8437	0.0039
Gadd45b	growth arrest and DNA-damage-inducible 45 beta	NM_008655	1.92	0.0000	1.08	0.6595	-1.13	0.7038	0.0000
Gadd45a	growth arrest and DNA damage inducible	NM_007836	1.66	0.0003	1.20	0.2245	-1.24	0.4200	0.0000
Por	NADPH cytochrome p450 oxidoreductase	NM_008898	1.66	0.0806	1.22	0.3847	1.04	0.9392	0.0402
Tieg1	TGFB inducible early growth response 1	NM_013692	-1.64	0.0011	-1.23	0.1983	-1.21	0.5440	0.0062
Gstm1	glutathione S-transferase mu 1	NM_010358	1.63	0.2258	-1.14	0.6339	-1.03	0.9622	0.0377
sult1c2	sulfotransferase family 1C, member 2	NM_026935	1.55	0.0044	1.16	0.3501	1.21	0.5440	0.0350
AhR	rat Ah receptor	NM_013464	1.48	0.0423	-1.05	0.7919	-1.12	0.7389	0.0039
Chk	choline kinase	NM_013490	-1.45	0.0041	-1.31	0.0876	1.02	0.9443	0.0039
Adcy6	adenylate cyclase 6	NM_007405	-1.43	0.0003	-1.20	0.1165	-1.05	0.7925	0.0004
Bhmt	betaine homocysteine methyl transferase	NM_016668	-1.40	0.2598	-1.16	0.4362	1.19	0.6392	0.0385
gstm2	glutathione S-transferase, mu 2	NM_008183	1.39	0.0313	-1.10	0.4647	-1.01	0.9754	0.0054
Dusp6	dual specificity phosphatase 6	NM_026268	1.39	0.0852	1.23	0.2007	-1.07	0.8288	0.0181
Lbp	Lipopolysaccharide binding protein	NM_008489	1.39	0.0104	1.15	0.2591	1.32	0.1249	0.0379
Rbp1	retinol binding protein 1, cellular	NM_011254	1.34	0.2364	-1.19	0.2941	1.00	0.9976	0.0101
Ephx1	epoxide hydrolase 1, microsomal; Eph1, Eph-1, AI195553	NM_010145	1.33	0.0486	-1.08	0.5597	1.05	0.8358	0.0181
Gsta4	Glutathione S-transferase, alpha 4	NM_010357	1.32	0.1312	-1.14	0.3506	-1.10	0.7389	0.0045
Ube2b	ubiquitin conjugating enzyme, E2B (RAD 6 homoly *S.cerevisea*)	NM_009458	-1.23	0.0358	-1.01	0.8808	1.00	0.9935	0.0350
Copeb	core promoter element binding protein	NM_011803	-1.21	0.8557	-1.28	0.1810	-1.44	0.1569	0.0471
Hao1	hydroxyacid oxidase	NM_010403	-1.20	0.8890	1.12	0.5641	1.38	0.4200	0.0402
Anpep	aminopeptidase N	NM_008486	1.20	0.4022	-1.20	0.1456	-1.05	0.8153	0.0070
Egfr	epidermal growth factor receptor	NM_007912	-1.18	0.7714	-1.04	0.7719	1.21	0.4200	0.0429
Gpam	glycerol-3-phosphate acyltransferase, mitochondrial	NM_008149	-1.16	0.2258	-1.00	0.9864	1.13	0.4200	0.0068
Olfr1161	olfactory receptor 1161	NM_146848	1.13	0.8471	1.30	0.0420	1.27	0.1191	0.0300
Keap1	Kelch-like ECH-assoc. protein 1, cytosolic inhibitor of NRF2)	NM_016679	-1.12	0.8821	-1.24	0.1165	-1.38	0.0941	0.0181
Ssrp1	Structure specific recognition protein 1	XM_130271	-1.10	0.8932	-1.13	0.3501	-1.34	0.1191	0.0121
mpg	N-methylpurine-DNA glycosylase	NM_010822	1.09	0.8894	1.30	0.0420	1.07	0.7269	0.0417
Ape2	AP endonuclease 2	NM_029943	-1.04	0.9530	-1.04	0.8232	-1.37	0.3673	0.0385
Ripk1	receptor (TNFRSF)-interacting serine-threonine kinase 1	NM_009068	-1.03	0.9565	-1.10	0.5255	-1.43	0.1191	0.0490
Nme2	expressed in non-metastatic cells 2, protein	NM_008705	1.02	0.9737	-1.24	0.2245	-1.12	0.7406	0.0238
Vegfb(2)	vascular endothelial growth factor B	NM_011697	-1.00	0.9944	-1.21	0.1279	-1.32	0.1191	0.0080

* Data ordered by fold-change in 100 mg/ml dose. There were no significant genes in 0.1 mg/ml dose. ^† ^Overall treatment effect

**Table 2:** ToxArray™ data normalized without EC: Overall adjusted p after exposure to PB and fold changes (relative to Control) and pairwise p-values at each dose for each gene (probe) that showed differential expression. Bold text indicates genes identified as differential overall (p < 0.05) in the EC-normalized analysis (Table 1).

			Control versus 100 mg/kg		Control versus 10 mg/kg		Control versus 1 mg/kg		James-Stein F-Test
			
Gene (probe) Name	Full name	GenBank	Fold change	Pairwise P-value	Fold change	Pairwise P-value	Fold change	Pairwise P-value	Overall P-value
Cyp2b9*	**cytochrome P450, 2b9, phenobarbitol inducible, type a, **	NM_010000	8.55	0.0000	3.56	0.0000	1.15	0.8412	0.0000
**Cyp2b10**	**Cytochrome P450, 2b10, phenobarbitol inducible, type b**	NM_009998	3.93	0.0000	2.92	0.0000	-1.08	0.8501	0.0000
**Gdf15**	**growth differentiation factor 15**	NM_011819	2.57	0.0000	1.26	0.6381	1.11	0.8311	0.0002
Gsta2	Glutathione S-transferase, alpha 2 (Yc2)	NM_008182	2.26	0.0353	1.08	0.9165	-1.22	0.7337	0.0160
**Gstm3**	**glutathione S- transferase mu 3 (EC 2.5.1.18) **	J03953	2.21	0.0000	-1.01	0.9793	1.31	0.4323	0.0002
**Gadd45b**	**growth arrest and DNA-damage-inducible 45 beta**	NM_008655	1.93	0.0000	1.04	0.8931	-1.12	0.6016	0.0000
**Lpin1**	**lipin 1, alias fatty liver dystrophy**	NM_015763	-1.66	0.0037	-1.02	0.9469	1.03	0.9303	0.0051
**Gadd45a**	**growth arrest and DNA damage inducible**	NM_007836	1.62	0.0024	1.25	0.4642	-1.22	0.4506	0.0000
Idi1	isopentenyl-diphosphate delta isomerase	NM_145360	-1.57	0.0131	-1.44	0.2098	-1.04	0.9206	0.0160
**Por**	**NADPH cytochrome p450 oxidoreductase**	NM_008898	1.55	0.0489	1.34	0.4157	1.07	0.8477	0.0341
**Rbp1**	**retinol binding protein 1, cellular**	NM_011254	1.49	0.0029	1.04	0.8850	1.14	0.5150	0.0094
**AhR**	**rat Ah receptor**	NM_013464	1.42	0.0676	1.06	0.8713	-1.07	0.8304	0.0436
**Dusp6**	**dual specificity phosphatase 6 **	NM_026268	1.41	0.0486	1.29	0.3460	-1.04	0.9018	0.0107
**Gsta4**	**Glutathione S-transferase, alpha 4**	NM_010357	1.41	0.0037	-1.00	0.9961	-1.09	0.6389	0.0006
**gstm2**	**glutathione S-transferase, mu 2**	NM_008183	1.40	0.0131	1.05	0.8594	1.07	0.7447	0.0329
**Ephx1**	**epoxide hydrolase 1, microsomal; Eph1, Eph-1, AI195553**	NM_010145	1.39	0.0004	1.08	0.6873	1.11	0.4882	0.0021
**Chk**	**choline kinase**	NM_013490	-1.39	0.0214	-1.17	0.5202	1.03	0.9165	0.0223
Ugt1a6	UDP glycosyltransferase 1 family, polypeptide A6	XM_135531	1.38	0.0019	1.22	0.2979	1.13	0.4624	0.0063
**Lbp**	**Lipopolysaccharide binding protein**	NM_008489	1.38	0.0254	1.12	0.6547	1.35	0.1677	0.0436
**Adcy6**	**adenylate cyclase 6**	NM_007405	-1.34	0.0037	-1.04	0.8511	1.01	0.9774	0.0068
Pitpn	phosphatidylinositol transfer protein	NM_008850	1.23	0.0000	1.07	0.4947	1.08	0.4311	0.0004
Olfr447	olfactory receptor 447	NM_146988	-1.20	0.6328	-1.19	0.4947	1.03	0.9112	0.0436
**Anpep**	**aminopeptidase N**	NM_008486	1.20	0.3851	-1.14	0.5202	-1.02	0.9253	0.0223
cd8b	CD8 antigen, beta chain	NM_009858	-1.16	0.0158	-1.07	0.5160	-1.11	0.2616	0.0436
Cyp2a4	Cytochrome P450, 2a4 (phenobarbitol inducer	NM_009997	1.14	0.3851	-1.07	0.6474	-1.09	0.4999	0.0045
Mmp9:	matrix metalloproteinase 9	NM_013599	-1.14	0.0735	-1.15	0.1425	-1.16	0.1632	0.0305
**Egfr**	**epidermal growth factor receptor**	NM_007912	-1.13	0.9847	1.07	0.8206	1.30	0.1971	0.0192
**Hao1**	**hydroxyacid oxidase**	NM_010403	-1.12	0.9867	1.24	0.5202	1.42	0.1991	0.0233
**Gpam**	**glycerol-3-phosphate acyltransferase, mitochondrial**	NM_008149	-1.10	0.9847	1.11	0.5202	1.20	0.1971	0.0063
**Ape2**	**AP endonuclease 2**	NM_029943	-1.09	0.9867	-1.18	0.5833	-1.43	0.1677	0.0395
Acadvl	acyl-Coenzyme A dehydrogenase, very long chain	NM_017366	-1.07	0.9867	1.10	0.5887	1.18	0.2294	0.0436
SCADfamilyprotein	mRNA for SCAD family protein	AJ296079	-1.05	0.9890	1.02	0.9388	1.26	0.1971	0.0395
PERP	PERP, TP53 apoptosis effector	NM_022032	-1.04	0.9890	1.06	0.7161	1.15	0.2892	0.0170
Acaa2	acetyl-Coenzyme A acyltransferase 2, mitochondrial	NM_177470	-1.02	0.9984	1.11	0.5202	1.24	0.1632	0.0094
**Vegfb(2)**	**vascular endothelial growth factor B**	NM_011697	-1.02	0.9984	-1.25	0.2731	-1.31	0.1677	0.0101
Fads2	fatty acid desaturase 2	NM_019699	-1.02	0.9984	1.22	0.3816	1.29	0.1902	0.0395
**Nme2**	**expressed in non-metastatic cells 2, protein**	NM_008705	1.01	0.9984	-1.21	0.4947	-1.14	0.5423	0.0436
Pxmp3	peroxisomal membrane protein 3	NM_008994	-1.01	0.9984	1.11	0.3446	1.14	0.1677	0.0242
Unc5h3	Unc5c:unc-5 homolog C (C. elegans), rostral cerebellar malform.	NM_009472	-1.00	0.9984	-1.08	0.6381	1.03	0.8466	0.0475

* Bolded text indicates genes also identified as differential in the EC-normalized analysis (Table 1)

**Table 3:** Real-time RT-PCR analysis of significant genes (p < 0.05) identified in microarray data normalized with and/or without EC dilution series.

Name	Full Name	GenBank	Overall Treatment Effect				Pairwise Comparisons						
			
			With EC	Without EC	# of Flagged Spots			With EC		Without EC		RT-PCR	
			p-value	p-value	Sample	Ref.	Comparison	foldchange	p-value	foldchange	p-value	foldchange	p-value
**Differentially expressed using both the EC and non-EC normalization**													

Adcy6	adenylate cyclase 6	NM_007405	0.0004	0.0068	6	0	100 mg/kg	-1.43	0.0003	-1.34	0.0037		
Chk	choline kinase	NM_013490	0.0039	0.0223	1	0	100 mg/kg	-1.45	0.0041	-1.39	0.0214	-2.39	0.0122
Cyp2b10	Cytochrome P450, 2b10, phenobarbitol inducible, type b	NM_009998	0.0000	0.0000	8	20	100 mg/kg	5.10	0.0000	3.93	0.0000	3.76	0.0001
Cyp2b10		NM_009998					10 mg/kg	2.95	0.0000	2.92	0.0000	1.04	0.8600
Cyp2b9	cytochrome P450, 2b9, phenobarbitol inducible, type a	NM_010000	0.0000	0.0000	15	22	100 mg/kg	8.29	0.0000	8.55	0.0000	3.04	0.0965
Cyp2b9		NM_010000					10 mg/kg	3.22	0.0000	3.56	0.0000	2.74	0.0052
Ephx1	epoxide hydrolase 1, microsomal; Eph1, Eph-1, AI195553	NM_010145	0.0181	0.0021	0	0	100 mg/kg	1.33	0.0486	1.39	0.0004	1.46	0.0350
Gadd45a	growth arrest and DNA damage inducible	NM_007836	0.0000	0.0000	17	0	100 mg/kg	1.66	0.0003	1.62	0.0024		
Gadd45b	growth arrest and DNA-damage-inducible 45 beta	NM_008655	0.0000	0.0000	25	1	100 mg/kg	1.92	0.0000	1.93	0.0000		
Gdf15	growth differentiation factor 15	NM_011819	0.0000	0.0002	10	1	100 mg/kg	2.83	0.0000	2.57	0.0000		
gstm2	glutathione S-transferase, mu 2	NM_008183	0.0054	0.0002	0	0	100 mg/kg	1.39	0.0313	1.40	0.0000	1.50	0.0013
Gstm3	glutathione S-transferase, mu 3	J03953	0.0000	0.0160	6	24	100 mg/kg	2.16	0.0000	2.21	0.0131		
Lbp	Lipopolysaccharide binding protein	NM_008489	0.0379	0.0051	1	25	100 mg/kg	1.39	0.0104	1.38	0.0037	1.60	0.0345
Lpin1	lipin 1	NM_015763	0.0039	0.0037	0	3	100 mg/kg	-1.94	0.0009	-1.66	0.0037		

**Differentially expressed using EC normalization only**													

AhR	rat Ah receptor	NM_013464	0.0039	0.0436	0	0	100 mg/kg	1.48	0.0423	1.42	0.0676	2.03	0.0003
Mpg	N-methylpurine-DNA glycosylase	NM_010822	0.041698	0.3782	25	25	10 mg/kg	1.30	0.0420	1.13	0.3911	1.42	0.0096
Olfr1161	olfactory receptor 1161	NM_146848	0.03003	0.0800	25	25	10 mg/kg	1.30	0.0420	1.12	0.3921	1.02	0.9618
sult1c2	sulfotransferase family 1C, member 2	NM_026935	0.0350	0.0572	18	25	100 mg/kg	1.55	0.0044	1.56	0.0214	1.60	0.0161
Tieg1	TGFB inducible early growth response 1	NM_013692	0.0062	0.1318	0	0	100 mg/kg	-1.64	0.0011	-1.45	0.0627	-2.88	0.0008
Ube2b	ubiquitin conjugating enzyme	NM_009458	0.0350	0.0897	0	0	100 mg/kg	-1.23	0.0358	-1.16	0.7402	-1.56	0.0058

**Differentially expressed using non-EC normalization only**													

cd8b	CD8 antigen, beta chain	NM_009858	0.2327	0.0436	25	25	100 mg/kg	-1.10	0.8557	-1.16	0.0158	1.40	0.3272
Dusp6	dual specificity phosphatase 6	NM_026268	0.0181	0.0107	16	0	100 mg/kg	1.39	0.0852	1.41	0.0486	1.40	0.0752
Gsta2	Glutathione S-transferase, alpha 2 (Yc2)	NM_008182	0.0681	0.0160	0	0	100 mg/kg	1.84	0.2849	2.26	0.0353	4.02	0.0026
Gsta4	Glutathione S-transferase, alpha 4	NM_010357	0.0045	0.0006	4	4	100 mg/kg	1.32	0.1312	1.41	0.0037	1.20	0.2200
Idi1	isopentenyl-diphosphate delta isomerase	NM_145360	0.0835	0.0436	0	0	100 mg/kg	-1.58	0.1234	-1.57	0.0254	-1.16	0.3523
Pitpn	phosphatidylinositol transfer protein	NM_008850	0.0969	0.0004	20	15	100 mg/kg	1.24	0.0624	1.23	0.0000	1.07	0.3100
Por	NADPH cytochrome p450 oxidoreductase	NM_008898	0.0402	0.0341	0	0	100 mg/kg	1.66	0.0806	1.55	0.0489	1.45	0.0064
Rbp1	retinol binding protein 1, cellular	NM_011254	0.0101	0.0094	0	7	100 mg/kg	1.34	0.2364	1.49	0.0029	1.13	0.3600
Ugt1a6	UDP glycosyltransferase 1 family, polypeptide A6	XM_135531	0.0942	0.0063	0	0	100 mg/kg	1.35	0.1044	1.38	0.0019	-1.06	0.4810

**Not differentially expressed**													

Anpep	aminopeptidase N	NM_008486	0.0070	0.0223	5	9	100 mg/kg	1.20	0.4022	1.20	0.3851		
Anpep		NM_008486					10 mg/kg	-1.20	0.1456	-1.14	0.9253	-1.04	0.6932
Cyp2a4	Cytochrome P450, 2a4 (phenobarbitol inducer)	NM_009997	0.0818	0.0045	24	24	100 mg/kg	1.19	0.2364	1.14	0.3851		
Cyp2a4		NM_009997					10 mg/kg	1.12	0.2511	-1.07	0.4999	1.32	0.0789
mpg	N-methylpurine-DNA glycosylase	NM_010822	0.0417	0.3782	25	25	100 mg/kg	1.09	0.8894	1.03	0.9890	-1.15	0.1757
Olfr1161	olfactory receptor 1161	NM_146848	0.0300	0.0800	25	25	100 mg/kg	1.13	0.8471	1.06	0.9867	-2.87	0.0386

**Table 4:** Criteria for determination of the true positives (TP), false negatives (FN), false positives (FP) and true negatives (TN) for the microarray results.

	Microarray Result using the Fs-test	
	**Positive**	**Negative**
Overall Treatment Effect	FDR p-value < 0.05	FDR p-value > 0.05
	**AND**	**OR**
Pairwise Comparison with Control*	FDR p-value < 0.05	FDR p-value > 0.05
**RT PCR Result**		
p-value < 0.05 (differentially expressed)	**TP**	**FN**
p-value > 0.05 (no change)	**FP**	**TN**

* comparison of individual doses against the control

**Table 5:** Measures of performance of microarray findings using normalization incorporating the external control features, compared to not incorporating the external control features.

A. Using all microarray pairwise comparisons				
	Microarray result using EC Normalization		Microarray result NOT using EC normalization	

RT-PCR result:	Significant	Not Significant	Significant	Not Significant
Significant	11 ^a^	3^b^	8^a^	9^b^
Not Significant	3^c^	10^d^	6^c^	4^d^
				
Sensitivity	78.6%		57.1%	
Specificity	76.9%		30.8%	
Positive Predictive Value	78.6%		47.1%	
Negative Predictive Value	76.9%		40.0%	
Efficiency of the test	77.8%		44.4%	

B. Using microarray pairwise comparisons that were in disagreement				

	Microarray result using EC Normalization		Microarray result NOT using EC normalization	

RT-PCR result:	Significant	Not Significant	Significant	Not Significant
Significant	5 ^a^	1^b^	2^a^	7^b^
Not Significant	3^c^	10^d^	6^c^	4^d^
				
Sensitivity	62.5%		25.0%	
Specificity	90.9%		36.4%	
Positive Predictive Value	83.3%		22.2%	
Negative Predictive Value	76.9%		40.0%	
Efficiency of the test	79.0%		31.6%	

Sensitivity= TP/(TP+FN); Specificity = TN/(TN+FP); Pos. Predictive Value= TP/(TP+FP); Neg. Predictive Value = TN/(TN+FN); Efficiency = (TP+TN)/(TP+TN+FP+FN).

^a ^True Positive; ^b ^False Negative; ^c ^False Positive; ^d ^True Negative
